# Exploring the impact of OSA on short-term survival in patients with AECOPD admitted to the ICU

**DOI:** 10.1371/journal.pone.0301646

**Published:** 2024-04-11

**Authors:** Liangfeng Liu, Yang Chen, Guanwen He, Bingbang Lin, Zhongshou Zhu, Rifu Wei, Yangbin Xu

**Affiliations:** 1 Department of Otolaryngology, Head and Neck Surgery, Ningde Municipal Hospital of Ningde Normal University, Ningde, Fujian, China; 2 Liverpool Centre for Cardiovascular Science at University of Liverpool, Liverpool John Moores University and Liverpool Heart & Chest Hospital, Liverpool, United Kingdom; 3 Fujian Medical University, Fuzhou, Fujian, China; AIIMS: All India Institute of Medical Sciences, INDIA

## Abstract

**Background:**

Acute exacerbation of chronic obstructive pulmonary disease (AECOPD) is characterized by a sudden worsening of chronic obstructive pulmonary disease (COPD) symptoms, which significantly contributes to hospitalizations related to COPD symptoms. Previous research has mainly focused on the correlation between obstructive sleep apnea (OSA) and COPD. However, there were few studies that investigated the short-term mortality rate of AECOPD patients with or without OSA.

**Methods:**

Data for our research was taken from the Medical Information Mart for Intensive Care Database IV. A total of 1332 patients were included in the study based on well-defined criteria for selection and exclusion. By analyzing the characteristics of AECOPD patients, we compared those with and without OSA.

**Results:**

There were 1122 AECOPD patients without OSA, 210 patients with OSA. In comparison to those without OSA, patients with OSA exhibited lower 30-day and 90-day ICU mortality with unadjusted HR, as well as lower hospital mortality with unadjusted OR. However, after adjustments were made, there were no significant associations observed between OSA and short-term mortality, including 30-day ICU mortality, 90-day ICU mortality, ICU mortality, and hospital mortality in AECOPD patients. Subgroup analysis revealed that OSA may act as a risk factor for AECOPD patients with a BMI lower than 30 kg/m^2^.

**Conclusions:**

There is no impact on short-term survival in AECOPD patients with OSA under intensive care unit (ICU) management and nursing.

## Introduction

Chronic obstructive pulmonary disease (COPD) is a major global health problem due to its high prevalence (about 10% of the adult population), rising incidence (related in part to the aging of the population) and very significant associated personal, social, and economic costs [1]. Despite the progress made in medical treatment, COPD remains the third leading cause of death worldwide [2]. Acute exacerbation of chronic obstructive pulmonary disease (AECOPD), characterized by sudden exacerbation of COPD symptoms, accounts for a significant number of COPD-related hospitalizations. Symptoms of AECOPD may include increased shortness of breath, dry mouth, lacking of energy, and increase in coughing [3]. AECOPD can be caused by a variety of factors, consisting of environmental exposures, viral or bacterial infections, and other triggers such as air pollution, allergens, and changes in weather [4–11]. Obstructive sleep apnea (OSA) is a sleep disorder characterized by breathing pauses or shallow breathing during sleep [12]. In the US, the occurrence of OSA is higher among men than women with 34% of males and 17% of females affected [13]. OSA commonly presents with symptoms such as loud snoring, daytime fatigue, excessive sleepiness during the day, and repeated pauses in breathing while asleep [14–16].

Patients suffering from COPD are prone to having OSA, which in turn increases the risk of AECOPD [17]. Prior studies have focused on the relationship between OSA and COPD, which can be challenging to manage when they coexist, adversely affecting the health of the individual [18, 19]. Given the relationship between OSA and COPD, we hypothesize that OSA will impact the short-term mortality of AECOPD patients.

Our retrospective cohort study, which used the Medical Information Mart for Intensive Care (MIMIC-IV) database, aimed to investigate the association between OSA and short-term mortality in patients hospitalized in the ICU with AECOPD.

## Material and methods

### Sources of data

Data for our research was taken from the Medical Information Mart for Intensive Care Database IV (MIMIC-IV, Version 2.0) [20], which was compiled between 2008 and 2019 and is available to the public. MIMIC-IV is an openly available database developed by the MIT Lab for Computational Physiology. The database is used to support research in areas such as critical care, medical informatics, epidemiology, and public health. MIMIC contains detailed information on patient demographics, vital signs, laboratory tests, medications, procedures, and outcomes. We accessed the database on October 3, 2023. We cannot identify individual participants since all the data had been stripped of identifying information.

### Patient and public involvement

The MIMIC-IV database received approval from the Institutional Review Board (IRB) of the Beth Israel Deaconess Medical Center (IRB Protocol #2001P001699) and was granted an exemption from the requirement for informed consent. Written informed consent for participation was not necessary for this study, in compliance with national legislation and institutional regulations.

### Selection of participants and stratification method

**Fig 1:**
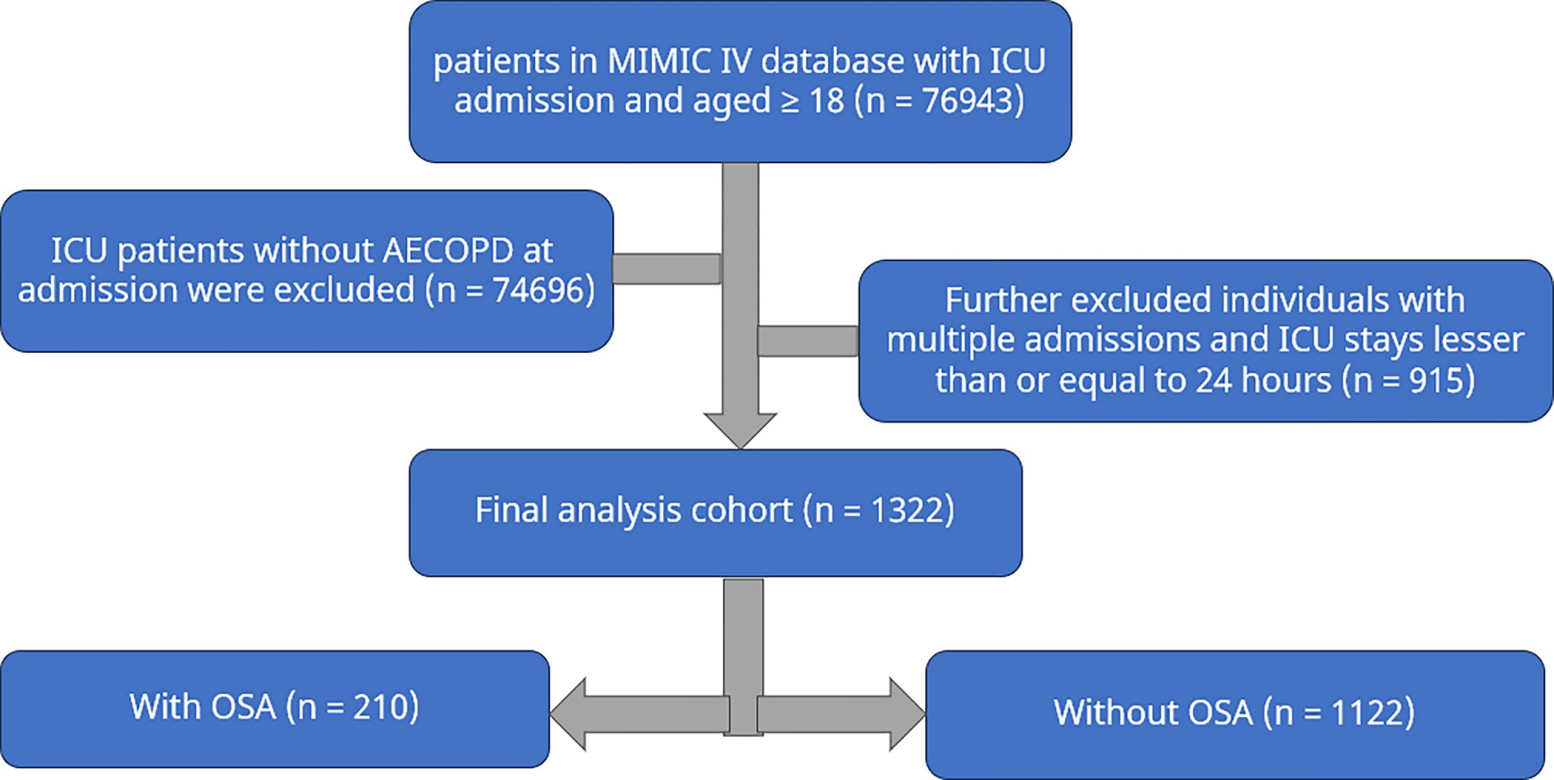
Flow diagram of study selection process. AECOPD, acute exacerbation of chronic obstructive pulmonary disease; ICU, intensive care unit; MIMIC IV, Medical Information Mart for Intensive Care IV; OSA, obstructive sleep apnea.

The diagnostic criteria were consistent with the International Classification of Diseases (ICD-9 and ICD-10). Disease coding for OSA is 32723(ICD-9) and G4733(ICD-10). Disease coding for AECOPD is 49121(ICD-9) and J440-441(ICD-10). All individuals admitted to the Intensive Care Unit(ICU) in this dataset were taken into account. The following were considered as exclusion criteria: In the dataset, a total of 76,943 participants were included. Firstly, individuals who were not diagnosed with AECOPD (n = 74696) were excluded. Additionally, those who had previously been admitted to the ICU (n = 296) were excluded, leaving a total of 1951 participants. Subsequently, individuals whose ICU stay was less than 24 hours (n = 340) and those who had been admitted to the hospital multiple times (n = 279) were also excluded. Finally, the study’s total patient count was ultimately decreased to 1332 individuals (Fig 1). The patients were categorized into two groups depending on whether they had OSA or not.

### Extraction of variables

We employed Structured Query Language to retrieve data for our study. The data collected consisted of various variables recorded within 24 hours of ICU admission, including age, sex, ethnicity, body mass index (BMI), vital signs, severity scores, comorbidities diagnosed using ICD-9 codes, laboratory results, and interventions administered. The detailed list of variables can be found in Table 1.

**Table 1:** Baseline characteristics of AECOPD patients on ICU admission.

Characteristics	All	Without OSA	With OSA	P-value
N	1332	1122	210	
Age (years)	72.52 ± 10.91	73.12 ± 10.88	69.32 ± 10.52	< 0.001
Male, n (%)	654 (49.1)	545 (48.6)	109 (51.9)	0.376
Body mass index, kg/m^2^	27.84 (23.07, 33.97)	26.94 (22.47, 32.12)	35.40 (29.85, 41.29)	< 0.001
Ethnicities,n (%)				0.621
White	945 (70.9)	799 (71.2)	146 (69.5)	
Non-white	387 (29.1)	323 (28.8)	64 (30.5)	
Vital Signs				
Heart rate (beat/min)	93.00 (80.00, 107.00)	94.00 (81.00, 108.25)	89.00 (79.00, 101.00)	<0.001
Mean blood pressure (mmHg)	83.00 (73.00, 95.00)	83.00 (73.00, 96.00)	82.00 (70.00, 93.00)	0.059
Respiratory rate (time/min)	22.00 (18.00, 26.00)	22.00 (18.00, 26.00)	20.00 (17.00, 24.00)	<0.001
Temperature (°C)	36.78 (36.50, 37.06)	36.78 (36.50, 37.06)	36.83 (36.50, 37.11)	0.343
Oxygen saturation (%)	96.00 (93.00, 99.00)	96.00 (93.00, 99.00)	96.00 (93.00, 99.00)	0.246
Severity score				
SOFA	1.00 (0.00, 2.00)	1.00 (0.00, 2.00)	1.00 (0.00, 2.00)	0.921
SAPS II	38.00 (30.00, 45.00)	38.00 (30.00, 46.00)	35.50 (27.00, 43.25)	0.002
OASIS	34.00 (28.00, 40.00)	34.00 (28.00, 41.00)	32.00 (27.00, 37.00)	<0.001
Comorbidities, n (%)				
Congestive heart failure	718 (53.9)	576 (51.3)	142 (67.6)	<0.001
Myocardial infarction	302 (22.7)	261 (23.3)	41 (19.5)	0.235
Cerebrovascular disease	110 (8.3)	92 (8.2)	18 (8.6)	0.857
Diabetes	430 (32.3)	326 (29.1)	104 (49.5)	<0.001
Liver disease	108 (8.1)	99 (8.8)	9 (4.3)	0.027
Paraplegia	33 (2.5)	29 (2.6)	4 (1.9)	0.561
Malignant cancer	199 (14.9)	178 (15.9)	21 (10.0)	0.029
Renal disease	345 (25.9)	277 (24.7)	68 (32.4)	0.020
Laboratory results				
Anion gap (%)	14.00 (12.00, 17.00)	14.00 (12.00, 17.00)	14.00 (12.00, 16.00)	0.140
Sodium (mmol/L)	139.00 (136.00, 142.00)	139.00 (136.00, 142.00)	140.00 (137.00, 142.00)	0.087
Potassium (mmol/L)	4.30 (3.90, 4.80)	4.30 (3.90, 4.80)	4.40 (4.00, 4.83)	0.053
Calcium (mg/dL)	8.60 (8.10, 9.00)	8.50 (8.00, 9.00)	8.70 (8.10, 9.10)	0.083
Creatinine (mg/dL)	1.00 (0.70, 1.50)	1.00 (0.70, 1.50)	1.10 (0.80, 1.60)	0.011
Blood urea nitrogen (mg/dL)	23.00 (16.00, 36.00)	24.00 (16.00, 36.00)	22.50 (16.75, 40.00)	0.395
Bicarbonate (mmol/L)	26.00 (23.00, 30.00)	26.00 (23.00, 30.00)	29.00 (24.00, 33.00)	<0.001
Chloride (mmol/L)	101.00 (97.00, 105.00)	101.00 (97.00, 105.00)	99.00 (96.00, 104.00)	0.005
White blood cell (×10^3^/μL)	10.60 (7.50, 14.80)	10.80 (7.60, 15.10)	9.65 (7.07, 13.60)	0.003
Platelet (×10^3^/μL)	209.00 (153.25, 271.00)	209.00 (154.00, 274.00)	207.00 (152.75, 265.00)	0.679
Red cell distribution width (%)	15.00 (13.90, 16.50)	14.90 (13.80, 16.40)	15.50 (14.40, 17.10)	<0.001
Red blood cell (m/μL)	3.68 (3.16, 4.22)	3.68 (3.15, 4.22)	3.62 (3.20, 4.22)	0.857
Mean red blood cell volume (fl)	93.00 (88.00, 97.00)	93.00 (88.75, 97.00)	92.00 (87.00, 98.00)	0.545
Interventions (1^st^ 24 h), n (%)				
Invasive mechanical ventilation	486 (36.5)	419 (37.3)	67 (31.9)	0.133
Non-invasive mechanical ventilation	138 (10.4)	92 (8.2)	46 (21.9)	<0.001
Dialysis	39 (2.9)	33 (2.9)	6 (2.9)	0.947
Vasopressor	335 (25.2)	294 (26.2)	41 (19.5)	0.041

Notes: Data are expressed as mean ± SD, median (interquartile range), or n (%). Students’ t-test (or the Kruskal-Wallis test) and Chi-square (or Fisher’s exact) tests were used for comparisons among groups. Statistical significance (*P* < 0.05).

Abbreviations: AECOPD, acute exacerbation of chronic obstructive pulmonary diseases; ICU, intensive care unit; OASIS, Overall Anxiety Severity and Impairment Scale; OSA, obstructive sleep apnea; SAPS II, Simplified Acute Physiology Score II; SD, standard deviation; SOFA, Sequential Organ Failure Assessment.

### Outcome variables

We collected several outcome variables, which included all-cause mortality rates within 30 days of ICU admission, within 90 days of ICU admission, during ICU stay, and during hospital stay, as well as the length of ICU and hospital stays. Among the six variables, all-cause 30-day and 90-day ICU mortality were the primary endpoints, while the others were secondary endpoints. We confirmed the 30 or 90-day ICU mortality rates by examining the death records in the database.

### Statistical analysis

The present study employed descriptive statistics to analyze the patient characteristics. For continuous data with normal distribution and homogeneity of variance, Student’s t-test was utilized, and the mean and standard deviation (SD) were reported. The Wilcoxon rank-sum test was utilized for continuous variables that lacked normal distribution and homogeneity of variance, and the resulting median and interquartile range (IQR) were reported. Categorical variables were presented as percentages and numbers, and comparisons were performed using either Pearson’s chi-squared test or Fisher’s exact test. Kaplan-Meier survival analysis was conducted to compare the distribution of all-cause 30-day or 90-day mortality between the two groups following ICU admission.

To evaluate the association between OSA and short-term mortality of individuals suffering from AECOPD, we employed logistic regression analysis to assess the association between OSA and mortality during ICU and hospital stays, and used Cox regression analysis to assess the association between OSA and mortality within 30 or 90 days of ICU admission. We used two separate models to control for potential confounders. Model 1 did not include any adjustments. Model 2 adjusted for age, gender, ethnicity, and body mass index. Model 3 included the same variables as Model 2, as well as additional variables, such as Sequential Organ Failure Assessment (SOFA) score, Simplified Acute Physiology Score II (SAPS II), Oxford Acute Severity of Illness Score (OASIS), congestive heart failure, myocardial infarction, cerebrovascular disease, diabetes, liver disease, paraplegia, malignant cancer, and renal disease.

To uncover potential effect modifiers, we also conducted a subgroup analysis. Patients was divided into different subgroups according to gender, age, BMI, congestive heart failure, diabetes, myocardial infarction, renal disease. In every subgroup, multivariate Cox or Logistic regression analyses was conducted based on the model 3.

## Results

### Baseline characteristics of the study population

The MIMIC-IV database had 2247 patients who met the earlier mentioned criteria for AECOPD. From these patients, only 1951 were included due to their first ICU admission, while 340 patients with less than 24 hours ICU stay and 279 with multiple hospital admissions were excluded. As a result, 1332 patients were enrolled in the study (Fig 1).

The study included 1122 patients without OSA and 210 patients with OSA (Table 1). The patients without OSA were older than those with OSA (*P* < 0.001). The sex (*P* = 0.376) and ethnicity (*P* = 0.621) of the two groups did not differ significantly. Patients without OSA had a lower BMI than those with OSA (*P* < 0.001). The heart rate (*P* < 0.001) and respiratory rate (*P* < 0.001) showed notable differences between the groups, whereas mean blood pressure (*P* = 0.059), temperature (*P* = 0.343), and oxygen saturation (*P* = 0.246) did not. The SAPS II (*P* = 0.002) and OASIS (*P* < 0.001) scales exhibited a significant variation in severity score between the groups, however, the SOFA scale (*P* = 0.921) did not show any significant variation. The two groups displayed variation in comorbidities, including congestive heart failure (*P*<0.001), diabetes (*P*<0.001), liver disease (*P* = 0.027), malignant cancer (*P* = 0.029), and renal disease (*P* = 0.020). Additionally, some laboratory results showed significant variation between the groups, including creatinine (*P* = 0.011), bicarbonate (*P* < 0.001), chloride (*P* = 0.005), white blood cell (*P* = 0.003), and red cell distribution width (*P* < 0.001). Furthermore, the proportion of patients who opted for non-invasive mechanical ventilation was notably higher in the OSA group (*P* < 0.001).

### ICU outcomes of the study population

The study revealed that 20.4% of the patients died within 30 days of their admission to the ICU. Patients with OSA had a lower mortality rate than those without OSA after 30 days of admission (*P* = 0.002). There were similar trends for 90-day ICU mortality and hospital mortality, but no significant variations were observed in ICU mortality or the median length of stay at the ICU and hospital between the two groups. More detailed information is presented in Table 2.

**Table 2:** ICU outcomes of AECOPD patients.

Outcomes	Without OSA	With OSA	P-value
Mortality, n (%)			
30-day after ICU admission	246 (21.9)	26 (12.4)	0.002
90-day after ICU admission	353 (31.5)	47 (22.4)	0.008
Stay at ICU	112 (10.0)	12 (5.7)	0.051
Stay at hospital	165 (14.7)	17 (8.1)	0.010
Length of stay, (days)			
Stay at ICU	3.00 (1.79, 5.98)	3.18 (1.77, 6.24)	0.589
Stay at hospital	8.74 (5.19, 14.88)	8.69 (5.57, 15.34)	0.905

Notes: Data are expressed as median (interquartile ranges) or n (%). Kruskal-Wallis test and Chi-square (or Fisher’s exact) tests were used for comparisons among groups. Statistical significance (*P* < 0.05).

Abbreviations: AECOPD, acute exacerbation of chronic obstructive pulmonary diseases; ICU, intensive care unit; OSA, obstructive sleep apnea.

**Fig 2:**
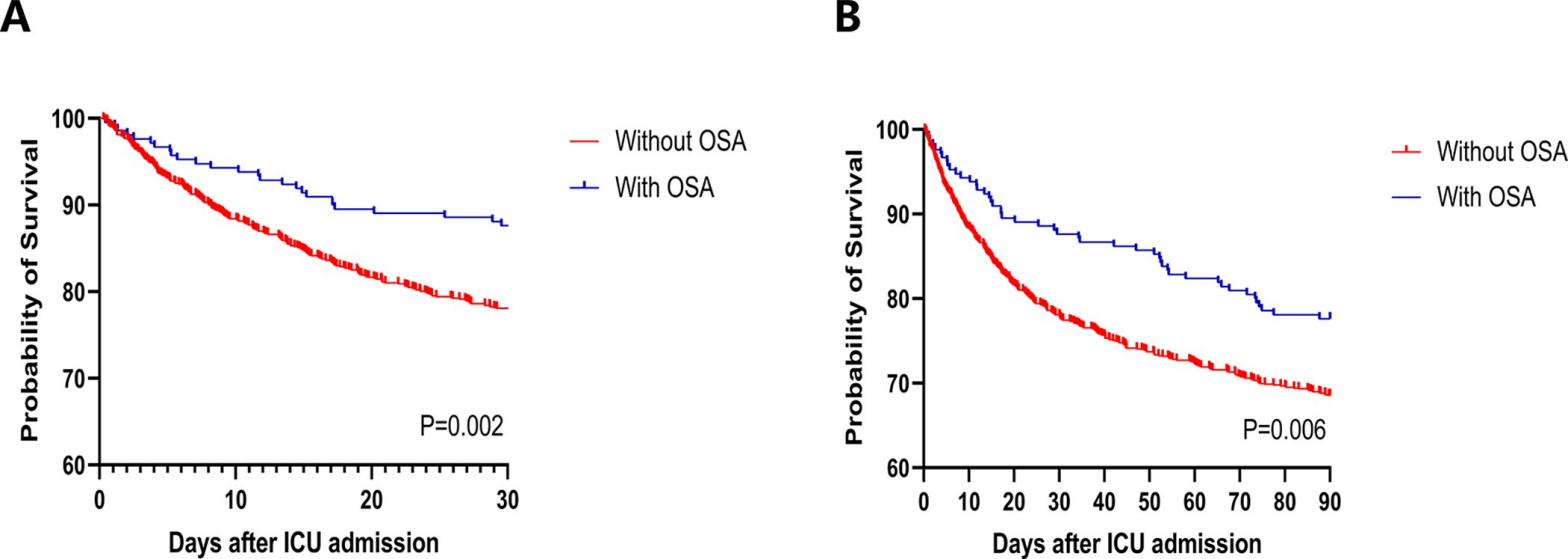
Kaplan–Meier survival analysis curves for 30-day (A) and 90-day (B) ICU mortality. ICU, intensive care unit; OSA, obstructive sleep apnea.

Fig 2 displays the Kaplan-Meier curves representing all-cause mortality rates at 30 or 90 days after admission to the ICU for both patient groups. These curves provide a clear visual representation of the mortality distribution, indicating that patients with OSA had a better chance of survival than those without OSA (*P* < 0.05).

### Associations between OSA and short-term mortality in patients with AECOPD

The study utilized multivariable Cox and logistic regression analysis to investigate the association between OSA and short-term mortality in AECOPD patients. The findings revealed that patients with OSA had lower 30-day (*P* = 0.002, HR = 0.534, 95% CI 0.357 to 0.800) and 90-day ICU mortality (*P* = 0.007. HR = 0.657, 95% CI 0.484 to 0.890), as well as reduced hospital mortality (*P* = 0.012, OR = 0.511, 95% CI 0.303 to 0.862) when compared to those without OSA, without adjusting for any factors. However, after adjusting for model 2 and model 3, no significant association was found between OSA and short-term mortality (including 30-day ICU mortality, 90-day ICU mortality, ICU mortality, and hospital mortality) in patients with AECOPD. The detailed results are presented in Table 3.

**Table 3:** Multivariate Cox or logistic regression analyses for OSA and outcomes in ICU patients with AECOPD.

Outcomes	HR or OR	95% CI	P-value
30-day ICU mortality			
Model 1	0.534	0.357, 0.800	0.002
Model 2	0.781	0.512, 1.192	0.252
Model 3	0.814	0.528, 1.253	0.349
90-day ICU mortality			
Model 1	0.657	0.484, 0.890	0.007
Model 2	0.894	0.649, 1.231	0.492
Model 3	0.918	0.662, 1.272	0.605
ICU mortality			
Model 1	0.547	0.296, 1.010	0.054
Model 2	0.706	0.371, 1.345	0.290
Model 3	0.809	0.397, 1.649	0.559
Hospital mortality			
Model 1	0.511	0.303, 0.862	0.012
Model 2	0.683	0.395, 1.184	0.174
Model 3	0.783	0.429, 1.429	0.426

Notes: Model 1: OSA without adjustment.

Model 2: Model 1 adjusted by age, gender, ethnicities, body mass index.

Model 3: Model 2 adjusted by Sequential Organ Failure Assessment score, Simplified Acute Physiology Score II, Overall Anxiety Severity and Impairment Scale score, congestive heart failure, myocardial infarction, cerebrovascular disease, diabetes, liver disease, paraplegia, malignant cancer, renal disease. Statistical significance (*P* < 0.05).

Abbreviations: AECOPD, acute exacerbation of chronic obstructive pulmonary diseases; CI, confidence interval; ICU, intensive care unit; HR, hazard ratio; OR, odds ratio; OSA, obstructive sleep apnea.

### Subgroup analysis

The study revealed that the impact of OSA differed significantly based on age, BMI, and diabetes status. Among patients with a BMI below 30 kg/m^2^, OSA was found to increase the risk of 90-day ICU mortality. However, no noteworthy association was observed between OSA and mortality in patients with a BMI above 30 kg/m^2^. For further information, please refer to Table 4.

**Table 4:** Multivariate Cox or logistic regression analyses for OSA and outcomes in AECOPD ICU patients in different subgroups according to the fully adjusted model (Model 3).

Subgroups	Outcomes [HR/OR (95% CI), P-value]							
	30-day ICU mortality	P value for interaction	90-day ICU mortality	P value for interaction	ICU mortality	P value for interaction	Hospital mortality	P value for interaction
Gender (male)	0.695 (0.365, 1.324), 0.269	0.515	0.825 (0.517, 1.317), 0.420	0.477	0.631 (0.228, 1.744), 0.375	0.639	0.753 (0.720, 1.773), 0.516	0.910
Gender (female)	0.925 (0.501, 1.710), 0.804		1.016 (0.632, 1.633), 0.947		1.059 (0.382, 2.938), 0.912		0.864 (0.367, 2.034), 0.738	
Age > 65 years	0.966 (0.617, 1.512), 0.879	0.045	1.054 (0.750, 1.482), 0.761	0.235	1.026 (0.490, 2.148), 0.945	0.100	0.905 (0.477, 1.717), 0.761	0.202
Age ≤ 65 years	0.224 (0.042, 1.188), 0.079		0.395 (0.118, 1.324), 0.132		0.134 (0.008, 2.142), 0.155		0.417 (0.063, 2.772), 0.365	
BMI < 30 kg/m^2^	1.466 (0.800, 2.687), 0.216	0.042	1.609 (1.002, 2.583), 0.049	0.024	1.189 (0.387, 3.650), 0.763	0.309	1.457 (0.595, 3.569), 0.410	0.072
BMI ≥ 30 kg/m^2^	0.610 (0.333, 1.117), 0.109		0.689 (0.442, 1.073), 0.100		0.690 (0.272, 1.745), 0.433		0.567 (0.252, 1.274), 0.170	
CHF (-)	0.383 (0.136, 1.085), 0.071	0.090	0.680 (0.356, 1.299), 0.243	0.269	0.400 (0.076, 2.114), 0.281	0.306	0.303 (0.063, 1.465), 0.137	0.098
CHF (+)	1.033 (0.632, 1.688), 0.897		1.017 (0.690, 1.499), 0.931		1.103 (0.489, 2.490), 0.813		1.036 (0.525, 2.046), 0.918	
Diabetes (-)	1.017 (0.581, 1.779), 0.953	0.244	1.016 (0.650, 1.586), 0.945	0.498	1.088 (0.420, 2.819), 0.863	0.513	0.940 (0.414, 2.138), 0.080	0.718
Diabetes (+)	0.634 (0.320, 1.253), 0.190		0.859 (0.529, 1.394), 0.538		0.623 (0.218, 1.784), 0.378		0.606 (0.243, 1.515), 0.284	
MI (-)	0.949 (0.581, 1.550), 0.834	0.361	1.048 (0.723, 1.518), 0.804	0.264	0.968 (0.436, 2.146), 0.306	0.361	0.865 (0.430, 1.740), 0.685	0.608
MI (+)	0.548 (0.211, 1.425), 0.218		0.628 (0.303, 1.299), 0.210		0.482 (0.091, 2.561), 0.392		0.596 (0.170, 2.092), 0.420	
Renal disease (-)	0.772 (0.434, 1.372), 0.377	0.920	0.827 (0.534, 1.279), 0.393	0.600	0.605 (0.232, 1.583), 0.306	0.406	0.655 (0.297, 1.443), 0.294	0.567
Renal disease (+)	0.963 (0.481, 1.925), 0.914		1.163 (0.688, 1.965), 0.574		1.723 (0.527, 5.630), 0.368		1.202 (0.444, 3.252), 0.717	

Notes: Model 3: OSA adjusted by age, gender, ethnicities, BMI, Sequential Organ Failure Assessment score, Simplified Acute Physiology Score II, Overall Anxiety Severity and Impairment Scale score, congestive heart failure, myocardial infarction, cerebrovascular disease, diabetes, liver disease, paraplegia, malignant cancer, renal disease. Statistical significance (*P* < 0.05).

Abbreviations: AECOPD, acute exacerbation of chronic obstructive pulmonary diseases; BMI, body mass index; CHF, congestive heart failure; CI, confidence interval; ICU, intensive care unit; HR, hazard ratio; OR, odds ratio; OSA, obstructive sleep apnea; MI, myocardial infarction.

## Discussion

This study used the MIMIC-IV database to explore the association between OSA and short-term mortality in AECOPD patients. The survival curves and mortality rates at 30 and 90 days for both groups demonstrated that AECOPD patients with OSA had significantly lower short-term mortality rates than those without OSA. However, after taking covariates into account, no statistically significant associations were discovered between OSA and short-term mortality, such as ICU mortality, hospital mortality, 30-day ICU mortality, or 90-day ICU mortality.

Upon comparing the baseline characteristics of both sets of patients, we observed that the AECOPD patients without OSA were significantly older than those with OSA. This finding aligns with a prior study suggesting that OSA may exacerbate the progression of age-related diseases such as COPD [21]. Obesity is a well-known risk factor for obstructive sleep apnea, and the prevalence of OSA increases with higher BMI [22]. Therefore, it is expected that AECOPD patients without OSA tend to have a lower BMI. This is consistent with our results. The SAPS II and OASIS scores for AECOPD patients without OSA were higher, indicating that these patients were more severely ill upon admission to the ICU. However, there was no significant difference in the SOFA scale, which may be due to the detection effectiveness of the three scales being different [23, 24]. Previous research has suggested that those suffering from both OSA and AECOPD are more likely to require invasive positive pressure ventilation [25], and our results confirm this viewpoint. Invasive positive pressure ventilation may resolve upper airway obstruction and eliminate the impact of OSA on AECOPD.

After adjusting for covariates, we found no correlation between OSA and short-term mortality in AECOPD patients, which differs from previous studies [17]. The coexistence of OSA and COPD, known as overlap syndrome [26]. Studies have indicated that individuals with a history of exacerbations are 1.436 to 1.485 times more likely to have OSA [17]. The presence of OSA can elevate an individual’s risk for developing AECOPD as a result of hypoxia, inflammation, compromise of the immune system, and modifications to the microbiota [17]. Moreover, there is evidence suggesting that OSA may increase the likelihood or severity of AECOPD. In a retrospective study, it was discovered that an increased risk of OSA correlated with a greater frequency of AECOPD and higher mortality rates or the first instance of hospitalization [27]. Hirayama et al. found that AECOPD patients who had OSA were more likely to require invasive positive pressure ventilation and spent a prolonged period in the hospital compared to those without OSA [25]. The risk of AECOPD and mortality may be lowered through the use of continuous positive airway pressure treatment [28]. However, our study reached a conclusion that totally differed from the results of previous studies. A possible explanation is that our data was derived from hospitalized patients in the ICU, and it is possible that certain treatments administered to these patients may have mitigated the impact of OSA on AECOPD. Further investigations in this direction are warranted in the future.

The subgroup analysis showed that the correlation between OSA and short-term mortality in AECOPD patients varied between different BMI subgroups. For AECOPD patients with a BMI lower than 30 kg/m^2^, OSA could be a risk factor. Previous research [29] indicating that overweight patients with a BMI of 25.0 kg/m^2^ or above, or those with moderate BMI, had a reduced risk for AECOPD compared to patients with a normal or low BMI, may explain these findings. There was also a weak interaction between OSA and age, but OSA was not significantly associated with short-term mortality in either of the specific two age subgroups.

A high mortality rate was observed in our study, despite the exclusion of the patients who had previously been admitted to the ICU for AECOPD. In UK, in-hospital mortality in patients admitted to hospital with an exacerbation of COPD was 4%; a further 3% died within 30 days of discharge, in 2014 [30]. In a meta-analysis of 6 studies, the average in-hospital mortality rate was 6.7% [31]. 20% died within 30 days from admission in our study. It is well-known that patients hospitalized in the ICU have severe illnesses and multiple comorbidities, which may contribute to a high mortality rate.

Although the data did not directly report the incidence of hypercapnic respiratory failure, the indicator of bicarbonate can indirectly reflect changes in respiratory function. Hypercapnic respiratory failure is a condition characterized by elevated levels of carbon dioxide in the blood. In the case of hypercapnic respiratory failure, the body attempts to compensate for the elevated carbon dioxide levels by increasing the concentration of bicarbonate in the blood. In our study, the Bicarbonate levels in the OSA group were significantly higher than those in the Without OSA group, consistent with previous research findings [32, 33]. Based on this result, we can reasonably infer that the incidence of hypercapnic respiratory failure in the OSA group may be higher than that in the Without OSA group. Hypercapnic respiratory failure is associated with a higher mortality rate [34]. However, our results showed no significant difference in short-term mortality rates between the two groups, which may be related to the higher use of non-invasive mechanical ventilation in the OSA group [35].

This study has several limitations. Firstly, like other retrospective designs, completely eliminating the impact of residual confounding variables is challenging. Secondly, it is not possible to establish a causal relationship between OSA and short-term mortality in AECOPD patients. Lastly, since not all variables are tracked in MIMIC-IV, certain indicators such as spirometry data for the diagnosis of COPD and polysomnography for the diagnosis of OSA are missing which may have an impact on our results.

## Conclusions

The most significant finding in this study is that OSA does not impact the short-term mortality of AECOPD patients admitted to the ICU. While previous research has explored the association between OSA and COPD, very few studies have looked into the influence of OSA on AECOPD patients. It remained unclear whether AECOPD patients with OSA required specialized attention and management. In this study, we tried to clarify the relationship between OSA and short-term mortality of AECOPD patients. We conclude that there is no impact on short-term survival in AECOPD patients with OSA under ICU management and nursing.

## Supporting information

S1 Checklist Human participants research checklist. (DOCX)
